# Discriminating malignancy of pulmonary nodules using a peripheral blood leukocyte DNA methylation-based SEMO network model

**DOI:** 10.3389/fonc.2026.1968970

**Published:** 2026-10-28

**Authors:** Bingyang Huang, Luyan Shen, Fengling Hu, Junjun Zhou, Xiaoyong Song, Zhongxing Dong, Jihua Wu, Hao Fu, Jinhui Feng, Zhuofan Yang, Guangyu Bai, Xiaowei Zhang, Jiafeng Shao, Lu Li, Jianghui Xiong, Shaohua Ma

**Affiliations:** 1 Department of Thoracic Surgery, Key Laboratory of Carcinogenesis and Translational Research (Ministry of Education/Beijing), Peking University Cancer Hospital and Institute, Beijing, China; 2 The Ninth Medical Center of Chinese Chinese People’s Liberation Army (PLA) General Hospital, Beijing, China; 3 DeepoMe Limited, Beijing, China; 4 Department of Thoracic Surgery, State Key Laboratory of Molecular Oncology, Beijing Key Laboratory of Carcinogenesis and Translational Research, Peking University Cancer Hospital and Institute, Beijing, China

**Keywords:** DNA methylation, ground-glass nodules, lung adenocarcinoma, peripheral blood leukocyte, risk stratification, SEMO network model, subcentimeter pulmonary nodules

## Abstract

**Background:**

Accurately discriminating malignant pulmonary nodules from benign lesions remains challenging, particularly in subcentimeter lesions. This study developed and internally validated a peripheral blood leukocyte (PBL) DNA methylation-derived Selective Epigenomic Module Optimization (SEMO) network model to improve risk stratification.

**Methods:**

A prospective specimen collection and retrospective analysis were performed. Preoperative PBL samples from 149 patients (102 were subcentimeter lesions) with surgically resected and pathologically confirmed pulmonary nodules were analyzed via targeted methylation sequencing of 500 aging/immune-related gene promoters. Protein-protein interaction (PPI) sets and functional pathway-based gene sets were integrated to derive SEMO features by calculating differential β-value distributions. Three models including clinical-radiological Baseline, SEMO-only, and Combined classifiers were established using elastic-net regression. Model performance was internally validated using 1,000 stratified bootstrap replicates with optimism correction and patient-level out-of-bag (OOB) prediction, no external validation was performed. Clinical utility was evaluated via decision curve analysis (DCA).

**Results:**

The SEMO-only and Combined models demonstrated comparable discrimination, with an apparent AUC of 0.996 (95% CI 0.988-1.000) and 0.993 (95% CI 0.978-1.000). Bootstrap internal validation yielded optimism-corrected AUCs of 0.966 and 0.966, with the more conservative patient-level OOB AUCs were 0.905 (95% CI 0.837–0.956) and 0.921 (95% CI 0.859–0.966). The calibration intercept, calibration slope, and Brier score were 0.019, 0.954, and 0.065 for SEMO-only model and -0.454, 1.390, and 0.076 for Combined model. In an exploratory analysis, nodules were stratified by predicted probability into low-risk (<0.37), intermediate-risk (0.37–0.87), and high-risk (≥0.87) categories. The subcentimeter evaluation as a prespecified exploratory endpoint, SEMO-only and Combined models maintained AUCs of 0.920 and 0.931.

**Conclusion:**

The PBL-derived SEMO model may serve as a non-invasive preoperative risk-stratification tool for patients with CT-detected nodules highly suspicious for malignancy who are being considered for surgery. External validation is required before clinical implementation.

## Introduction

Lung cancer remains the leading cause of cancer-related mortality worldwide, with approximately 2.5 million new cases and 1.8 million deaths annually (1). Although comprehensive treatment modalities, including surgical resection, chemotherapy, radiotherapy, targeted therapy, and immunotherapy, have significantly improved patient outcomes, the overall 5-year survival rate remains low (2). The prognosis of lung cancer patients is closely associated with the disease stage, earlier stages correlating with higher survival rates, early detection is paramount. The widespread adoption of low-dose computed tomography (LDCT) (3, 4) has markedly increased the detection of indeterminate pulmonary nodules, of which 9–30% are pathologically confirmed to be benign upon surgical resection (5–8). Crucially, the vast majority of these screen-detected lesions are micronodules (<5 mm) or small nodules (5–10 mm) (5, 9, 10). Owing to their small size and atypical features, accurately assessing their malignancy risk poses a significant clinical challenge. Established clinical–radiological models, including the Mayo Clinic (11), Veterans Affairs (12), Brock (13) and Peking University People’s Hospital (PKUPH) (14)models, estimate malignancy risk from patient characteristics and nodule morphology, with reported AUCs ranging from 0.79 to >0.90 in their development cohorts (**Supplementary Table S1**). These models were derived from screening or incidental solid nodule populations, and rely largely on nodule size and morphological features that are least discriminative in small and ground-glass lesions. Current management strategies are unsatisfactory: frequent CT surveillance exposes patients to cumulative radiation and sustained anxiety, whereas invasive biopsy or resection may subject patients whose nodules prove benign to unnecessary surgical trauma and the associated operative morbidity, psychological distress, and healthcare costs (9, 10, 15). CT-based preoperative risk assessment alone remains insufficiently accurate for these lesions. Consequently, there is an urgent clinical imperative to develop novel non-invasive diagnostic approaches capable of preoperative risk re-stratification of CT-suspected high-risk nodules.

Aberrant DNA methylation is a well-established early hallmark of adenocarcinoma, often preceding atypical adenomatous hyperplasia (16–19). Cancer-specific alterations in DNA methylation can be detected in the sputum (20), bronchoalveolar lavage fluid (21), and pleural effusions (22) of lung cancer patients. Although plasma cell-free DNA (cfDNA) methylation tests were more widely used and show promise for early detection, their clinical specificity is often compromised by low tumor-fraction abundance, high fragmentation, and normal DNA contamination (23, 24). Conversely, cancer-specific methylation alterations in peripheral blood leukocytes reflect systemic immune responses, offering a complementary diagnostic approach (25, 26). For instance, a peripheral blood mononuclear cell (PBMC)-based model utilizing a combination of *NCOA2*, *RUNX3*, and *BTD* with age and sex effectively distinguished healthy individuals from lung cancer patients, with an AUC of 0.914, and an AUC of 0.917 for subcentimeter adenocarcinoma (27). However, this study lacked a benign pulmonary nodule control group, precluding evaluation of the model’s true performance in distinguishing benign from malignant nodules. In addition, the complex procedures required for PBMC isolation hinder widespread clinical application. Because the clinical need for non-invasive benign–malignant differentiation is greatest for small nodules, which are difficult to biopsy and frequently indeterminate on imaging, we prespecified an evaluation of model performance in the subcentimeter subgroup.

Furthermore, traditional cfDNA or immune cell methylation models typically rely on statistical “multi-gene” or “few-gene” panels (23, 27–29). These approaches have several limitations: (i) they rely on simple statistical correlations between individual genes and malignancy without functional context; (ii) they treat genes as independent features, ignoring the intergenic interactions and the cumulative pathway effects, compromising model robustness and biological interpretability; (iii) their statistical significance frequently fluctuates across different cohorts and technological platforms, weakening the generalizability (29, 30). Network biology addresses these issues by modeling complex molecular interactions to capture the pathophysiological essence of diseases, thereby offering superior clinical predictability (31–33). Previously, we developed the SEMO algorithm to quantify functional perturbations within protein interaction networks, validating its predictive performance in clinical cohorts of COVID-19 infections (34). In that formulation, each feature paired a hub’s interaction neighborhood with a chemical-target gene set, and the framework was accordingly termed selective remodeling of protein networks. The present work leaves the index unchanged but pairs each hub neighborhood with a functional pathway gene set; the acronym is therefore expanded as Selective Epigenomic Module Optimization throughout this article. Aging and cancer converge on a shared set of meta-hallmarks, among them genomic instability, epigenetic alteration, and chronic inflammation, providing a biological rationale for interrogating aging- and immune-related loci in circulating leukocytes (35). Herein, we performed DNA methylation profiling of a 500 aging/immune-related genes panel to characterize the epigenetic landscape of peripheral blood leukocyte in 149 patients with pulmonary nodules. To address our small and imbalanced cohort, we utilized a robust workflow featuring stratified nested cross-validation paired with elastic-net logistic regression, validated via 1,000 stratified bootstrap repetitions. Finally, we constructed and compared a clinical-radiologic baseline model against an integrated model combining baseline features with SEMO signatures to evaluate the added diagnostic value of the SEMO network features.

## Methods

### Study participants​

Peripheral blood samples and clinical data were collected prospectively and analyzed retrospectively. This study initially enrolled 178 patients who underwent chest CT examinations revealing pulmonary nodules (≤30 mm) highly suspicious for lung cancer and subsequently received surgical resection between November 2019 and January 2021 at the Ninth Medical Center of the Chinese PLA General Hospital. All participants were over 18 years of age. Exclusion criteria are listed in **Figure 1**. A total of 29 patients were excluded from enrollment for the following reasons: receipt of antitumor treatment prior to or at the time of blood collection (n= 4), presence of infection or immune system disease (n = 7), inadequate sample quality (n = 17), and small cell lung cancer (n = 1). 149 patients were included in the final analysis, and the subcentimeter (n=102) analysis is exploratory subgroup analysis. Preoperatively, 10 mL of venous blood was collected in EDTA anticoagulant tubes. Peripheral blood leukocytes (PBLs) were isolated within 4 hours via centrifugation and stored at -80 °C until DNA extraction. This study was approved by the Ethics Committee of the Ninth Medical Center of the Chinese PLA General Hospital (K2018 Ethics Committee No. 01) and Peking University Cancer Hospital (2024YJZ75-GZ01). Written informed consent for the de-identified retrospective secondary analysis was waived. The study was registered (ChiCTR2600119035) and was conducted in accordance with the Declaration of Helsinki.

**Figure 1:**
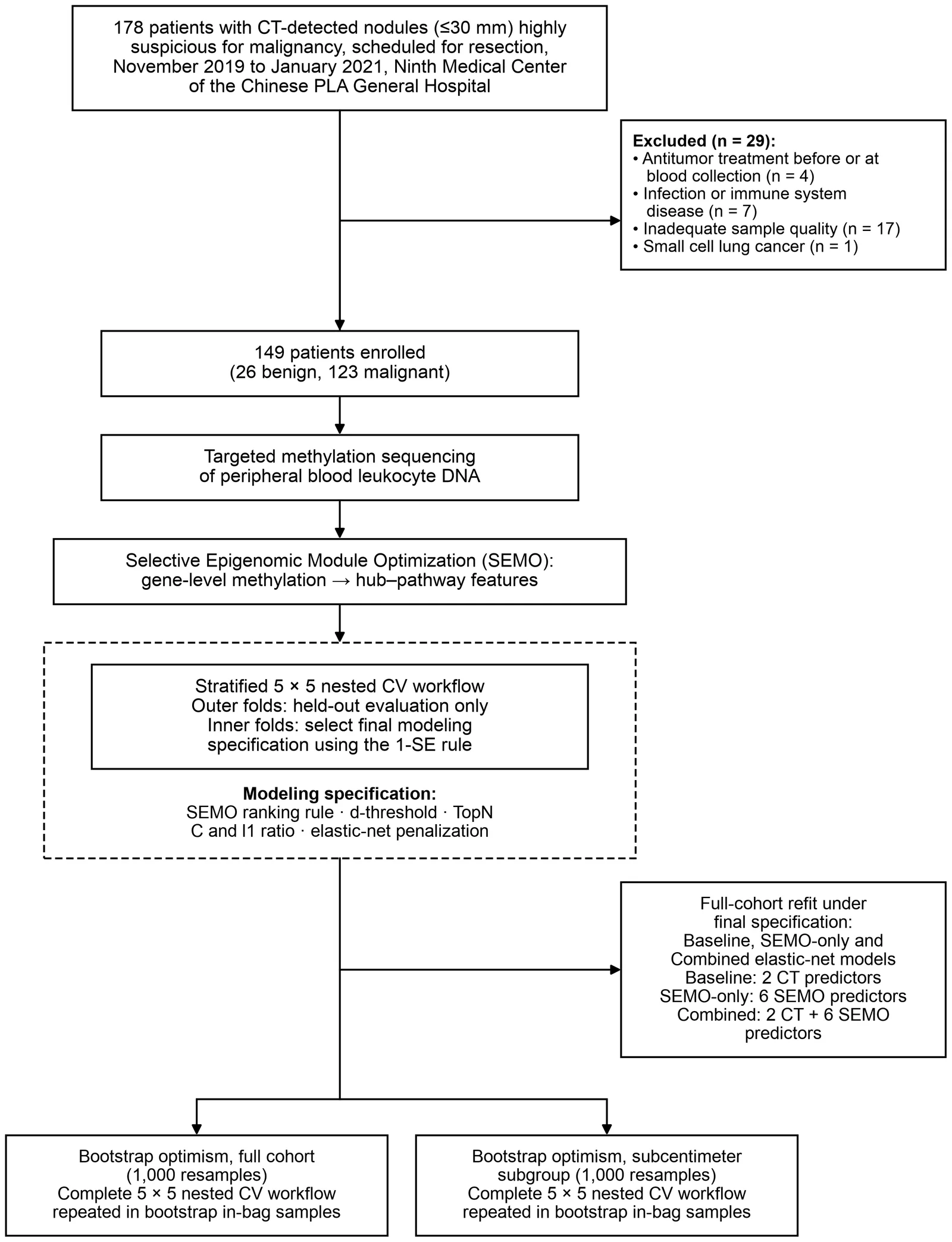
Study flowchart and analysis workflow.

### PBL DNA-methylation profiling

In this study, the CapoMe methylation detection kit (from DeepoMe Co., Ltd.) was utilized to perform high-throughput sequencing on the Q-T7B sequencer (BGI Genomics Co., Ltd., Shenzhen, China) to analyze DNA methylation patterns of PBLs from patients with pulmonary nodules. This kit was originally designed to detect the promoter methylation levels across a panel of 500 genes, enabling multidimensional assessment of aging (e.g., metabolic, immune, and organ-specific aging). For each gene, the average β-value of all CpG sites within its promoter region was calculated as its methylation signature value, where a higher β-value indicates an elevated degree of methylation.

### Construction of network-based biomarkers with SEMO

The SEMO algorithm was employed to quantify network-level epigenetic imbalance between benign and malignant nodules. First, protein-protein interaction (PPI) data were retrieved from the Human Protein Reference Database (HPRD, http://www.hprd.org) Release 9 (13 April 2010) (36). HPRD contains only experimentally validated, manually curated interactions, which keeps false-positive edges out of the seed network at the cost of coverage, a trade-off that suits a method which partitions the network into hub neighborhoods, and the same database underlies the earlier work from which the present approach derives (34). The global PPI network was then partitioned into distinct hub gene sets, each centered on a protein encoded by one of the 500 genes in our panel. A hub gene set “i.n” was defined as all genes interacting with protein i, n is a label, not a count. For example, “p53.n” denotes the set of all interaction partners of p53, and the size of a hub gene set is the number of interacting genes it contains. As in that prior work (34), only hub sets containing ≥20 interacting genes were retained for subsequent analysis. To identify SEMO features associated with nodule malignancy, we systematically screened multiple functional annotation and pathway databases, including Gene Ontology, KEGG, Reactome, BIOCARTA, WikiPathway, Hallmark gene sets, and the Pathway Interaction Database. The computational workflow for extracting SEMO features is illustrated in **Figure 2**. For a given PPI gene set i containing multiple genes, its intersection with a specific functional pathway gene set k was denoted as X, while the difference set (genes in PPI gene set i that do not belong to pathway k) was denoted as Y.

**Figure 2:**
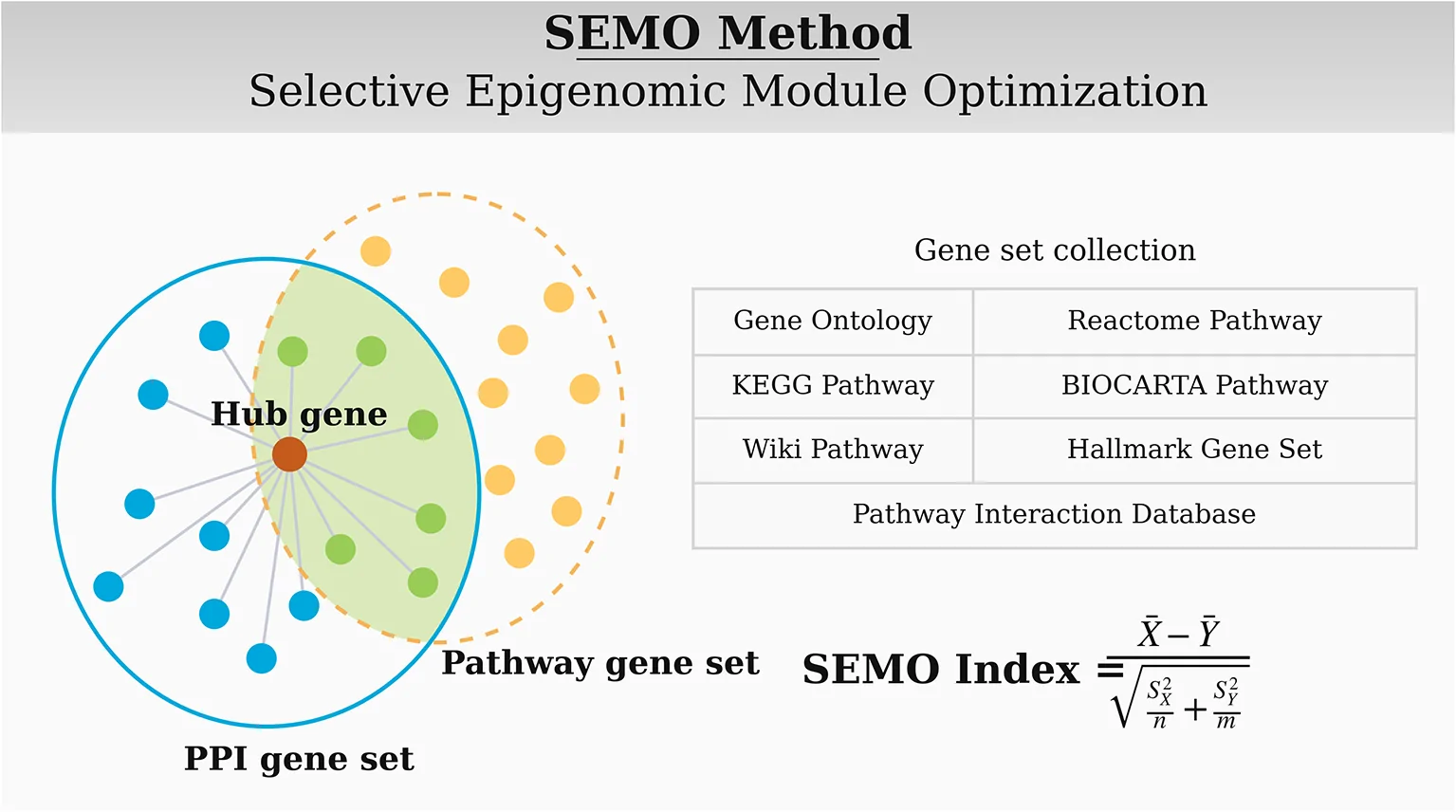
Construction of SEMO features. For each hub-centered PPI gene set i and functional pathway gene set k, the intersection between the two sets was defined as set X, whereas the remaining genes in the same PPI neighborhood that were not included in pathway k were defined as set Y. The SEMO index was calculated by comparing the mean promoter methylation β values between sets X and Y, normalized by their corresponding variances and set sizes. Each hub-pathway combination was formatted as “hub gene–pathway name” and treated as one SEMO feature.

We calculated the mean β-value and variances for both gene sets X and Y, where n and m represent the number of genes in sets X and Y, respectively. For any combination of PPI gene set i and pathway gene set k, the calculated SEMO index was defined as follows (Equation 1):

(1) $$T_{ik}=\frac{\bar{x}-\bar{y}}{\sqrt{\frac{s_{x}^{2}}{n}+\frac{s_{y}^{2}}{m}}}$$

where $\bar{x}$ and $\bar{y}$ are the mean promoter β values of sets X and Y, and $s_{x}^{2}$ and $s_{y}^{2}$ their sample variances, was defined as a SEMO feature and formatted as “PPI set name - pathway name”(e.g.,”CCK.n-GO_DEVELOPMENTAL_GROWTH”). Tik is algebraically identical to Welch’s two-sample t statistic contrasting gene sets X and Y, it therefore quantifies the methylation imbalance between the portion of a hub neighborhood that falls inside a given pathway and the portion that falls outside it. A hub–pathway pair was admitted only when the two gene sets overlapped more than expected by chance, assessed by a hypergeometric test with a threshold of P< 0.01; pairs failing this criterion were not evaluated. The SEMO feature index was computed for each patient across all predefined combinations. Feature significance was evaluated using a unequal-variance (Welch) t test comparing benign and malignant groups. Finally, candidate SEMO features were ranked across the full cohort and the top 500 features were prioritized. The P values served only to rank features for a fixed Top-N selection and were therefore not adjusted for multiple testing.

### Development and optimization of diagnostic models

To evaluate the diagnostic utility of SEMO features, we developed three predictive frameworks: a Baseline model (clinical-radiological), a SEMO-only model, and an integrated model. The clinical–radiological variables comprised nodule size, nodule type (pure ground-glass, heterogeneous ground-glass, part-solid, solid and small solid), spiculation, boundary clarity, upper-lobe location, emphysema, age, sex, and four smoking variables (ever-smoker status, smoking years, cigarettes per day and years since quitting). To address the constraints of our small and imbalanced cohort, we utilized elastic-net penalized logistic regression, which combines *L1* (Lasso) and *L2* (Ridge) regularization to perform simultaneous feature selection and mitigate multicollinearity (37, 38). The hyperparameter optimization space encompassed SEMO ranking methods (*P*-value vs. dlogp, dlogp is a composite ranking score computed as |SMD| × (−log10 p), where SMD is the standardized mean difference between benign and malignant groups and p is the two-sample t-test P value, such that features with both large effect size and small P value are ranked highest), effect size thresholds (d-threshold), feature selection limits (TopN), penalization strength (C), and l1 ratio. To ensure unbiased performance estimation, a stratified 5 × 5 nested cross-validation (CV) scheme (39) was implemented to audit the entire model-building workflow. Within each inner loop, missing-value imputation, standardization, categorical encoding, SEMO ranking, Top-N selection, and hyperparameter tuning were executed exclusively on the training folds. To prevent overfitting, the simplest model configuration within one standard error (1-SE) of the maximum achieved area under the receiver operating characteristic curve (ROC-AUC) was selected. The final models were subsequently refitted on the entire cohort using the optimized hyperparameter specifications. The development, internal validation and cross-validation procedures followed established prediction-model development guidance (40).

### Internal validation and subgroup analysis

Internal validation was performed using 1,000 stratified bootstrap replicates (41). In each replicate, the complete pipeline, including stratified 5 × 5 nested-CV hyperparameter tuning and 1-SE rule, was re-executed. Optimism for ROC-AUC, precision-recall AUC (PR-AUC), and Brier scores was estimated by contrasting performance between the bootstrap datasets and the original cohort, yielding optimism-corrected estimates. Three estimates are reported. (i) The apparent estimate is the performance of the final model measured on the same 149 patients used to fit it. (ii) The classic bootstrap optimism-corrected estimate subtracts the mean optimism from the apparent estimate, optimism being the performance of each bootstrap model on its own bootstrap sample minus the performance of that same model on the original cohort; this corrects the apparent value but still uses every patient for both fitting and evaluation. (iii) The patient-level pooled out-of-bag (OOB) estimate assigns each patient a single predicted probability averaged only over those bootstrap replicates in which that patient was not sampled, separately, the distribution of per-replicate OOB AUCs is displayed to convey replicate-to-replicate variability. The OOB estimate never reuses a patient’s own data for fitting and is the most conservative of the three, and we report it as the primary measure of discrimination. It nevertheless remains an internal-validation estimate obtained in the same cohort, it does not escape the optimism introduced by the full-cohort reduction of the feature space described above, and it is neither equivalent to external validation.

For the prespecified exploratory analysis subcentimeter subgroup (nodules ≤10 mm), this complete bootstrap and optimism-correction workflow was repeated independently as an exploratory analysis.

As an exploratory analysis, a two-threshold risk-stratification scheme was derived for the SEMO-only model from out-of-bag predicted probabilities. The lower cut-point was fixed at the probability yielding 98% sensitivity (rule-out) and the upper cut-point at 90% target specificity (rule-in). Because both cut-points were derived from the same cohort, the resulting operating characteristics are apparent estimates and were not further optimism-corrected; they are reported for illustrative purposes and require external validation.

### Comparison with traditional risk models​

The performance of the SEMO-based models was benchmarked against four validated malignancy risk prediction models on the same cohort: the Mayo Clinic (11), Veterans Affairs (VA) (12), Brock (13), and Peking University People’s Hospital (PKUPH) (14) models. Each model was applied using its original published coefficients, without refitting or recalibration in our cohort; because no model parameters were estimated from these data, no optimism correction was applicable and their performance estimates represent direct application to the cohort. To ensure data integrity, predictors for these clinical models were independently extracted by two board-certified thoracic surgeons and one board-certified radiologist (each with more than 10 years of clinical experience), with discrepancies resolved via consensus.

### Statistical analysis

Statistical analyses were conducted using Python (version 3.9.6), leveraging NumPy, pandas, SciPy, scikit-learn, and statsmodels. Continuous variables were expressed as medians with interquartile ranges and compared via the Mann–Whitney U test. Categorical variables were presented as counts with percentages and evaluated using the chi-square test or Fisher’s exact test, as appropriate. Model performance was evaluated across three dimensions: discrimination (ROC-AUC and PR-AUC), calibration (plots, intercept, and slope) (42), and overall prediction error (Brier score) (43). Clinical utility was further assessed using decision-curve analysis across a range of threshold probabilities. Standardized net benefit was calculated as net benefit divided by disease prevalence, allowing interpretation on a 0–1 scale (44, 45). Because predictive values and net benefit depend on disease prevalence, a sensitivity analysis was performed at assumed malignancy prevalences of 70% and 90%, spanning the range reported for resected nodules (5–8), and of 5–30%, representing lower-prevalence CT-detected populations. Predictive values were recomputed from Bayes’ theorem with sensitivity and specificity held at their observed values, and net benefit was recomputed after updating the model intercept by log[odds(assumed prevalence)/odds(0.826)], which leaves the ranking of patients and discrimination unchanged. 95% CIs were calculated by the Wilson method for sensitivity and specificity, by the method of Mercaldo et al. for predictive values at assumed prevalences (46), and by 2,000 stratified bootstrap resamples for net benefit. All statistical tests were two-sided, with *P* value< 0.05 indicating statistical significance.

## Results

### Participants and clinical characteristics

The study cohort exclusively comprised surgical candidates, with 123 of 149 patients (82.6%) pathologically confirmed as malignant, predominantly consisting of Stage 0–I adenocarcinoma (95.9%). Because the benign group was small (n = 26; n = 23 in the subcentimeter subgroup), specificity-dependent estimates were imprecise, with 95% CIs approximately 20–35 percentage points wide (for example, a specificity of 92.3% has a 95% CI of 75.9–97.9%); 95% CIs are therefore reported for all such metrics. Prespecified exploratory subcentimeter nodules (≤10 mm) predominated in the cohort (n = 102, 68.5%), with a median overall nodule size of 8.0 mm (benign: 5.0 mm; malignant: 9.0 mm). Ground-glass opacity (GGO) was highly prevalent, occurring in 116 cases (77.9%). Notably, spiculation was significantly more frequent in malignant lesions (34.1% vs. 7.7%), whereas a family history of malignancy was more common in patients with non-malignant nodules (30.8% vs. 11.4%), reflecting selection effects within this surgery-enriched cohort. The non-malignant group primarily encompassed atypical adenomatous hyperplasia (AAH; n = 7), alveolar epithelial hyperplasia(n = 6), and sclerosing hemangioma (n = 13). To ensure homogeneous baseline profiles, all patients were confirmed to have normal complete blood counts and were screened to exclude systemic conditions that could alter peripheral blood leukocyte DNA methylation, such as inflammatory bowel disease, infections, autoimmune diseases, and hematologic disorders. Detailed clinical and demographic characteristics are summarized in **Table 1**.

**Table 1:** Patient characteristics.

Characteristic	Entire cohort (N = 149)	Benign (n = 26)	Malignant (n = 123)	P value
Age (years), median (IQR)	62.0 (51.0, 69.0)	60.0 (54.0, 65.0)	63.0 (50.0, 70.0)	0.413
Sex, no. (%)				1.000
Female	98 (65.8)	17 (65.4)	81 (65.9)	
Male	51 (34.2)	9 (34.6)	42 (34.1)	
Smoking history, no. (%)				
Ever smoked	32 (21.5)	7 (26.9)	25 (20.3)	0.630
Family history, no. (%)				
Any malignancy	22 (14.8)	8 (30.8)	14 (11.4)	0.029
Lung cancer	10 (6.8)	4 (15.4)	6 (5.0)	0.077
Personal history, no. (%)				
Any malignancy	23 (15.6)	3 (11.5)	20 (16.5)	0.767
Nodule characteristics				
Size (mm), median (IQR)	8.0 (5.0, 13.0)	5.0 (4.0, 7.0)	9.0 (5.5, 14.0)	<0.001
Size ≤10 mm, no. (%)	102 (68.5)	23 (88.5)	79 (64.2)	0.019
Ground-glass nodule, no. (%)	116 (77.9)	20 (76.9)	96 (78.0)	1.000
Upper-lobe location, no. (%)	90 (60.4)	14 (53.8)	76 (61.8)	0.595
Spiculation, no. (%)	44 (29.5)	2 (7.7)	42 (34.1)	0.008
Emphysema, no. (%)	36 (24.2)	5 (19.2)	31 (25.2)	0.719

### SEMO features generation and covariate independence

Using the SEMO algorithm, 19,832 candidate features were initially generated, from which the top 500 features ranked by benign–malignant t-test *P* values were selected to define the predictor space. These 500 SEMO features comprised combinations of 135 hub genes and 211 functional pathways. Notably, individual hub genes repeatedly partnered with multiple functional pathways, and conversely, distinct pathways recurrently clustered within the neighborhoods of different hub genes. This non-random convergence indicates that epigenetic variances between benign and malignant lesions were structured into recurrent, network-level functional modules rather than being discretely distributed. Consequently, the SEMO framework successfully reorganizes subtle, scattered methylation fluctuations into high-dimensional, topologically relevant features, providing stable and biologically interpretable signals for risk stratification.

Previous studies have reported that age (47, 48) and smoking (49) can influence DNA methylation profiles. To ensure these molecular signals were driven by malignancy rather than demographic or exposure-related modifiers, we evaluated the association between the top 500 SEMO features and key covariates (age, sex, smoking history, family history of any tumor, and family history of lung cancer) using Spearman rank correlation. In this exploratory assessment, no SEMO feature was significantly associated with these covariates after false discovery rate (FDR) correction (all q > 0.05; maximum |ρ| = 0.273), may suggest that the network signatures are not primarily driven by these clinical factors.

### Model optimization, performance, and clinical utility

Through stratified 5 × 5 nested cross-validation, the final modeling specifications were locked within the established framework. For the SEMO feature preselection, the optimal configuration was determined as *rank method* = dlogp, *d_thresh* = 0.0, and *TopN* = 500. Under the one-standard-error (1-SE) rule, the simplest hyperparameter configurations within one standard error of the peak AUC were selected to penalize overfitting. Specifically, the tuned Elastic-Net logistic regression models locked the *l1_ratio* at 0.1 across all frameworks, with penalization strengths (C) determined at 0.011288, 0.069519, and 0.037927 for the Baseline (clinical-radiological), SEMO-only, and Combined models, respectively. In the outer five-fold cross-validation analysis, predicted probabilities from the five held-out folds were pooled to construct the cross-validated ROC curves. The mean outer-fold AUCs achieved were 0.740 ± 0.133 (range: 0.563-0.928) for the Baseline model, 0.918 ± 0.021 (range: 0.896-0.951) for the SEMO-only model, and 0.907 ± 0.024 (range: 0.888-0.936) for the Combined model. For the Baseline model the mean of the five fold-specific AUCs (0.740) exceeds the AUC obtained by pooling the five folds’ held-out predictions (0.653). This divergence is expected when a model is unstable across folds and one class is rare: each outer fold contains only about five benign patients, so fold-specific AUCs are both highly variable (range 0.563–0.928) and computed on a fold-specific probability scale, whereas pooling requires predictions from different folds to be comparable on a common scale. The gap is therefore a symptom of between-fold instability of the clinical–radiological model. The SEMO-only and Combined models, whose fold-to-fold variability was roughly six-fold smaller (SD 0.021 and 0.024 versus 0.133), show almost no such gap. After determining the final specification, the elastic-net model was refitted on the full cohort to identify the final predictors with non-zero coefficients. Complete equations, coefficients, and scaling parameters are provided in the **Supplementary Materials** SEMO deployable formulas. The SEMO-only model captured six non-zero SEMO Hub–Pathway features:

ITCH__GO_UBIQUITIN_LIKE_PROTEIN_LIGASE_BINDING, 350
PRKACA__GO_POSITIVE_REGULATION_OF_LOCOMOTION, 351
AR__HP_ABNORMALITY_OF_LONG_BONE_MORPHOLOGY, 352
LPAR2__GO_CELL_GROWTH, 353
HDAC3__GO_CHROMATIN_BINDING, 354
ITPR1__GO_CATION_TRANSMEMBRANE_TRANSPORT

Bootstrap feature-selection frequencies robustly demonstrated the structural stability of the SEMO signatures, all six final SEMO predictors ranked among the ten most top-weight-recurrent features in both settings, and were selected with non-zero coefficients in a median of 99.3% of the 1,000 bootstrap replicates (range 92.9–99.7%) in the SEMO-only model and 98.2% (range 88.4–99.1%) in the Combined model. Four final SEMO predictors (ITCH-, AR-, PRKACA-, and HDAC3-related modules) consistently selected in >97% of the 1,000 bootstrap iterations in both the SEMO-only and Combined settings (**Supplementary Tables S2** and **Supplementary Tables S3**). The two CT descriptors (nodule size and spiculation) that appear in the final Combined model are those retained after elastic-net penalization. The Combined model incorporated these identical six SEMO features alongside two CT-derived radiological variables, forming an eight-predictor integrated model. In the full-cohort apparent analysis, the Baseline, SEMO-only, and Combined models yielded apparent AUCs of 0.754, 0.996, and 0.993, respectively; corresponding PR-AUCs were 0.930, 0.999, and 0.999, with Brier scores of 0.160, 0.036, and 0.053 (**Figures 3A, B**). These apparent estimates reflect optimal model fit, though they remain inherently susceptible to optimistic bias in small-sample, high-dimensional settings. Notably, the Combined model demonstrated no distinct apparent discrimination advantage over the SEMO-only model.

**Figure 3:**
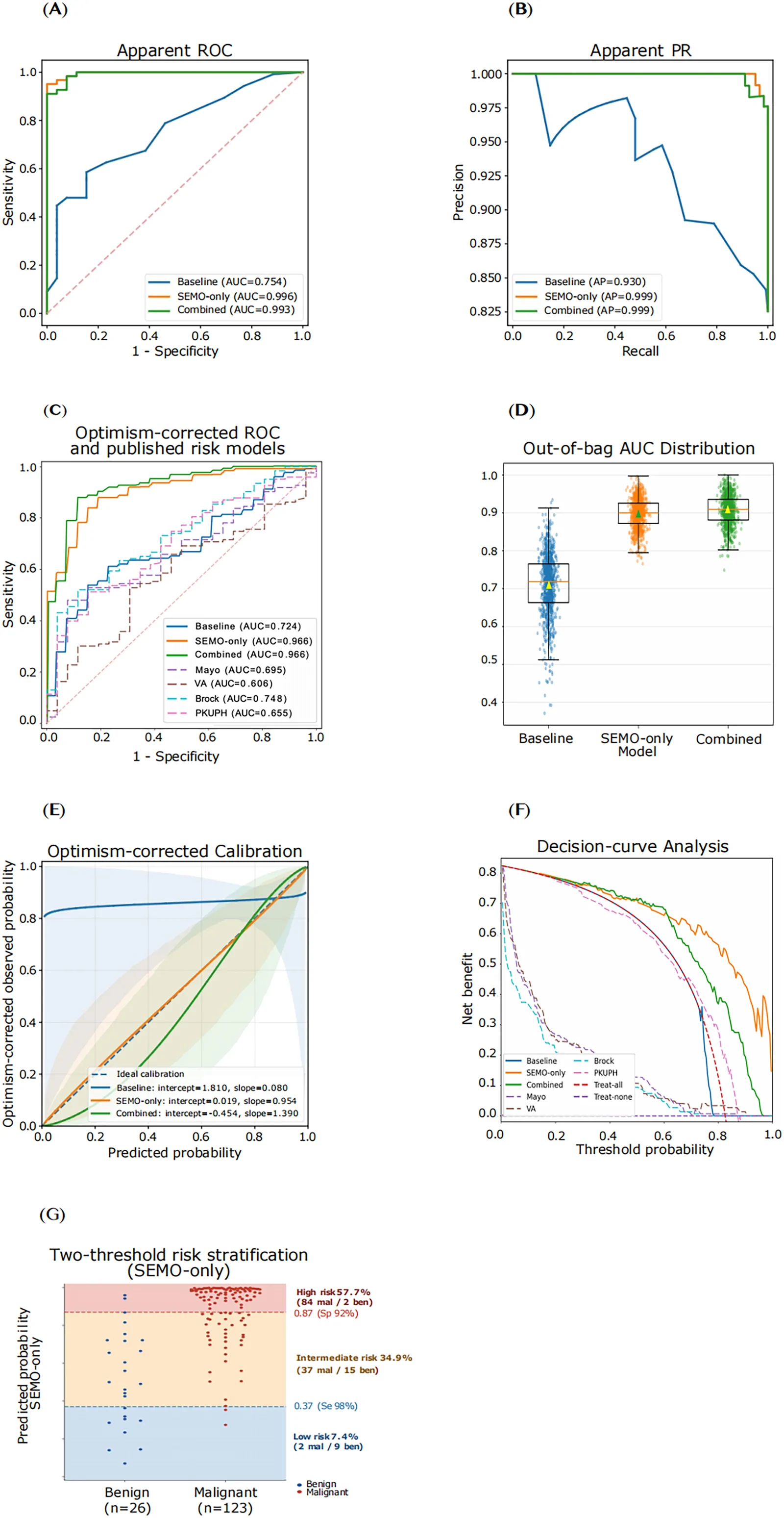
Full-cohort model performance and threshold-specific classification. **(A)** Apparent receiver operating characteristic curves for the Baseline, SEMO-only, and Combined models. **(B)** Apparent precision–recall curves for the three models. **(C)** Receiver operating characteristic curves for the three developed models shown together with four published clinical–radiological risk models (Mayo, VA, Brock, and PKUPH) applied to the same cohort. **(D)** Out-of-bag AUC distribution across bootstrap iterations. Each point represents the OOB AUC from one bootstrap iteration. **(E)** Bootstrap optimism-corrected calibration curves with calibration intercepts and slopes. **(F)** Decision-curve analysis across threshold probabilities of 0.01–0.80 for the Baseline, SEMO-only, and Combined models and for the four published risk models (Mayo, VA, Brock, and PKUPH), with the treat-all and treat-none strategies shown as references. Net benefit for the published models was derived from their original fixed coefficients without refitting. **(G)** Two-threshold risk stratification of the SEMO-only model. Positive classification indicated predicted malignant/high-risk status. ROC, receiver operating characteristic; PR, precision–recall; SEMO, Selective Epigenomic Module Optimization; VA, Veterans Affairs; PKUPH, Peking University People’s Hospital.

To correct for apparent overoptimism, 1,000 stratified bootstrap repetitions were performed (**Figure 3C**). The optimism-corrected AUCs were 0.724 (95% CI, 0.633–0.819) for the Baseline model, 0.966 (95% CI, 0.944–0.986) for the SEMO-only model, and 0.966 (95% CI, 0.943–0.984) for the Combined model, with corrected Brier scores of 0.160, 0.065, and 0.076, respectively (**Supplementary Table S4**). Two complementary out-of-bag (OOB) summaries are reported: the distribution of per-iteration OOB AUCs across bootstrap replicates (**Figure 3D**), and a single patient-level pooled OOB AUC, in which each patient’s predicted probability was derived only from bootstrap iterations where that patient was not included in model training. For the patient-level pooled OOB analysis, the Baseline, SEMO-only, and Combined models achieved AUCs of 0.683 (95% CI, 0.576–0.785), 0.905 (95% CI, 0.837–0.956), and 0.921 (95% CI, 0.859–0.966), respectively.

In the bootstrap corrected calibration analysis (**Figure 3E**), the Baseline model performed poorly, yielding an adjusted calibration intercept of 1.810 and a slope of 0.080. Conversely, the SEMO-only model demonstrated excellent calibration, with an adjusted intercept of 0.019 and a slope of 0.954 (approximating the ideal values of 0 and 1). The combined model showed greater calibration distortion (intercept = −0.454; slope = 1.390), reflecting under-dispersed and overly conservative predicted probabilities.

Decision-curve analysis (DCA) across threshold probabilities of 0.01–0.80 (**Figure 3F**) showed that the SEMO-only and Combined models provided the greatest net clinical benefit, remaining above all four traditional risk models (Mayo, VA, Brock, and PKUPH) and the default treat-all and treat-none strategies at thresholds above 0.1. Given the high malignant prevalence of this surgical cohort (82.6%), the treat-all reference itself yielded a high net benefit at low thresholds (0.806 at a threshold of 0.10). The decision curves therefore describe the pre-operative decision problem of this surgical cohort. Within the actionable range, the advantage of the SEMO-based models over the traditional models widened as the threshold increased: at a threshold of 0.40, the SEMO-only and Combined models achieved net benefits of 0.729 and 0.740, respectively, versus 0.676 for the best-performing traditional model (PKUPH), 0.157 for Mayo, 0.125 for VA, and 0.110 for Brock; at a threshold of 0.50, the corresponding values were 0.718 (SEMO-only) and 0.711 (Combined) versus 0.638 (PKUPH), 0.128 (Mayo), 0.107 (VA), and 0.094 (Brock). In contrast, none of the four traditional models exceeded the treat-all reference at low thresholds (all ≤0.806 at a threshold of 0.10 and ≤0.782 at 0.20), indicating no incremental clinical value over a default treat-all strategy in this cohort. The apparent proximity of the PKUPH curve to the treat-all line at low thresholds reflected its compressed predicted probabilities (median 0.98, with 81.9% of patients assigned a probability >0.8), which caused it to behave like a treat-all strategy rather than to provide genuine discrimination. The clinical-radiologic Baseline model tracked the treat-all reference closely until high thresholds before declining. Together, these findings indicate that only the peripheral-blood-leukocyte SEMO signatures translated into a consistent net clinical benefit over both default strategies and conventional risk models. As a prespecified exploratory analysis, we derived a two-threshold risk-stratification strategy for the SEMO-only model from out-of-bag predicted probabilities (**Figure 3G**). A lower threshold was defined at 98% sensitivity (to avoid missing malignant nodules) and a higher threshold at 90% specificity (to avoid misclassifying benign nodules as high risk), yielding cut-points of 0.37 and 0.87, respectively. 11 nodules (7.4%) were low risk (9 benign, 2 malignant; predicted probability<0.37), 52 (34.9%) were intermediate risk (15 benign, 37 malignant; 0.37–0.87), and 86 (57.7%) were high risk (2 benign, 84 malignant; ≥0.87) (**Figure 3G**; **Supplementary Table S5**). These risk categories correspond to a three-tier management pathway in which low-risk nodules undergo routine CT surveillance, intermediate-risk nodules are referred for multidisciplinary team (MDT) discussion and individualized follow-up, and only high-risk nodules proceed to surgical resection. Under this pathway, 24 of 26 (92.3%) patients with benign nodules would have been spared unnecessary resection and the associated surgical trauma, whereas all 26 underwent resection in this cohort. Of the 86 high-risk nodules allocated to resection, 84 were malignant on final pathology, yielding, at the cohort prevalence of 82.6%, a PPV of 97.7% (95% CI, 91.9–99.4), a sensitivity of 68.3% (95% CI, 59.6–75.9) and an NPV of 38.1% (95% CI, 27.1–50.4). At the 0.87 cut-point, the net benefit of this resection decision was 0.474 (95% CI, 0.319–0.597), compared with −0.342 for a resect-all strategy. Conversely, 39 of 123 (31.7%) malignant nodules were not allocated to immediate resection. Of these, 37 nodules were categorized as intermediate risk and referred for MDT discussion, their median diameter was 8.0 mm (IQR, 5.0–13.0), and 29 (78.4%) were ground-glass nodules, which generally follow an indolent course and the risk associated with deferring resection in this group is likely limited. The 0.37 cut-point had a sensitivity of 98.4% (95% CI, 94.3–99.6), a specificity of 34.6% (95% CI, 19.4–53.8) and an NPV of 81.8% (95% CI, 52.3–94.9). Both cut-points were imprecise, with bootstrap medians of 0.373 (95% interval, 0.273–0.552) and 0.889 (95% interval, 0.723–0.963) (**Supplementary Table S5**); operating characteristics at alternative cut-points spanning these intervals are given in **Supplementary Table S6**. Because predictive values depend on disease prevalence, they were recalculated at assumed malignancy rates, holding sensitivity and specificity constant (**Supplementary Tables S7** and **Supplementary Tables S8**; **Supplementary Figures S1** and **Supplementary Tables S2**). Across the 70–90% malignancy rate reported in surgical series, the PPV of the resection cut-point was 95.4% (95% CI, 84.5–98.7) at 70% and 98.8% (95% CI, 95.5–99.7) at 90%. Its net benefit remained above both the resect-all and resect-none strategies, at 0.402 (95% CI, 0.271–0.507) and 0.517 (95% CI, 0.348–0.651), respectively; the NPV of the 0.37 cut-point was 90.1% (95% CI, 67.7–97.5) and 70.3% (95% CI, 35.2–91.2), respectively. Because both cut-points were derived from the same cohort, these operating characteristics are apparent estimates and require external validation.

### Subgroup validation and benchmarking against traditional models

Exploratory subgroup analysis was conducted within the subcentimeter cohort by replicating the entire bootstrap-refitting pipeline, with 79 malignant and 23 benign patients. The Baseline model showed poor discrimination (AUC, 0.578; 95% CI, 0.452–0.696), whereas the SEMO-only and Combined models retained high optimism-corrected AUCs of 0.920 (95% CI, 0.855–0.970) and 0.931 (95% CI, 0.870–0.977), respectively (**Figure 4**), paired with robust PR-AUCs (0.976 and 0.978 vs. 0.832) and lower Brier scores (0.096 and 0.106 vs. 0.175).

**Figure 4:**
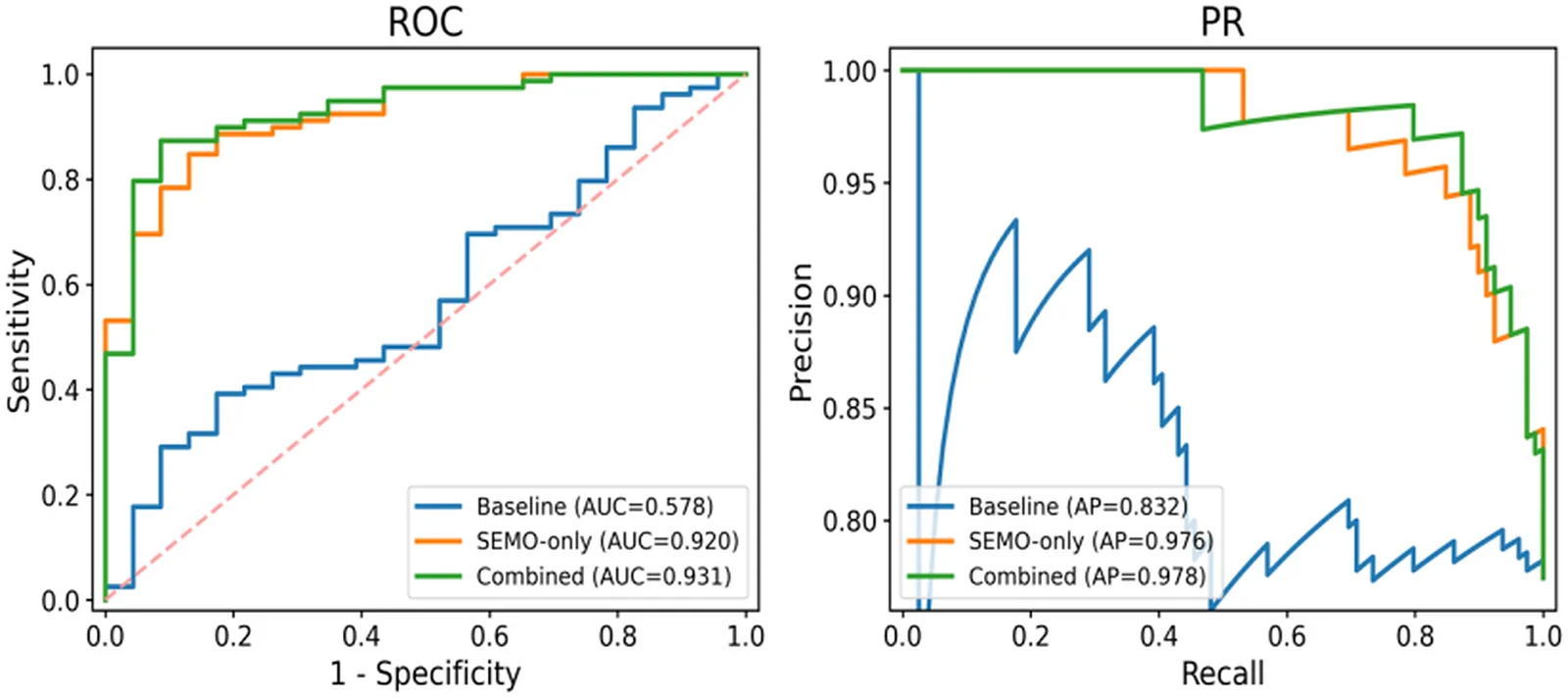
Model performance in the subcentimeter subgroup (≤10 mm).

Finally, we benchmarked our frameworks against four traditional clinical–radiological risk models (Mayo Clinic, Veterans Affairs, Brock, and PKUPH). Applied with their original published coefficients, these conventional models achieved AUCs of 0.695, 0.606, 0.748, and 0.655, respectively, in the full cohort (**Figure 3C**). These models were not fitted to our data, their estimates require no optimism correction; the comparison in **Figure 3C** therefore contrasts optimism-corrected estimates for the developed models with uncorrected estimates for the published models. Their sensitivities were also limited, ranging from 47.1% to 56.4%, despite relatively high specificities in some models. Within the critical subcentimeter subgroup, their diagnostic performance degraded substantially, yielding AUCs of 0.593, 0.469, 0.668, and 0.458, respectively (**Supplementary Tables S9**, **S10**). Their sensitivities ranged from 15.2% to 58.2%, indicating limited ability to identify malignant subcentimeter nodules in this cohort. Collectively, these findings highlight that network-level epigenetic signatures substantially outperformed established clinical risk models in this cohort, particularly in the challenging diagnosis of small and subcentimeter pulmonary nodules.

## Discussion

The widespread clinical application of LDCT has significantly increased the detection of subcentimeter and ground-glass pulmonary nodules, reshaping the landscape of early-stage lung cancer screening (3, 4, 50). However, this technological advancement introduces a profound clinical quandary. Conventional radiological features often lack the resolution and specificity required to accurately differentiate early-stage malignancies from benign or premalignant lesions, rendering risk assessment heavily reliant on clinician experience and prone to interobserver variability (51). While longitudinal CT surveillance mitigates the risk of missing a malignancy, it imposes cumulative radiation exposure, financial burden, and severe psychological anxiety among patients (52, 53). Invasive procedures such as needle biopsy or immediate surgical intervention carry localized procedural risks such as pneumothorax (54). Moreover, it suffers from non-negligible false-negative rates due to the potential of missing the target lesion. Clinicians are thus frequently suspended in a dilemma between overtreatment and undertreatment. This underscores an urgent clinical imperative for non-invasive, objective adjunctive tools capable of refining preoperative risk stratification and guiding individualized management.

DNA methylation alterations are early carcinogenic events widely explored for non-invasive cancer detection (16–19, 28). Earlier liquid biopsy strategies predominantly relied on few-gene cfDNA methylation panels (30), failing to capture the vast epigenetic heterogeneity of early lung adenocarcinoma, particularly in indolent or small lesions with minimal tumor DNA shedding. To overcome these sensitivity limitations, high-dimensional cfDNA methylation models were developed. For instance, the 100-feature PulmoSeek classifier achieved an overall AUC of 0.843, but its discriminative capacity significantly declined in 6–10 mm nodules (AUC 0.670; specificity 0.250) (23). Although its successor, PulmoSeek Plus, partially restored discrimination by integrating cfDNA methylation with clinical and radiological features for subcentimeter and subsolid lesions, with an elevated AUC of ~0.91 and high sensitivity of ≥99%, it still relies heavily on multimodal inputs (28). Moreover, these contemporary assays remain bound to tumor-derived cfDNA, inheriting inherent biological vulnerabilities alongside clinical barriers, including high assay costs and intense operational complexity (24, 55).

To address these bottlenecks, we leveraged PBL DNA methylation profiling rather than hunting scarce, highly fragmented tumor-derived cfDNA methylation. This approach capitalizes on the amplified, systemic host-immune epigenetic responses invoked at the very inception of tumorigenesis (27), ensuring robust signal detection even in subcentimeter nodules that exhibit minimal DNA shedding. Moreover, our framework abandons the conventional, fragmented strategy of utilizing “multi-gene” or “few-gene” lists based purely on superficial statistical associations. Instead, we developed the SEMO platform to extract network-level epigenetic signatures. By integrating PBL methylation data with protein-protein interaction (PPI) networks and functional biological pathways, SEMO reorganizes scattered, subtle methylation fluctuations into high-dimensional, topologically organized, and functionally correlated modules.

Importantly, whereas a prior study (27) predominantly compared lung cancer patients to healthy controls, our study evaluated a clinically relevant surgical cohort with pathologically verified benign lesions rather than a healthy population. This design directly challenges the SEMO platform to differentiate malignancy-specific host immunity from the confounding, broad-spectrum immune noise triggered by benign lung pathologies. Notably, the SEMO-only model achieved diagnostic discrimination on par with the integrated Combined model (both optimism-corrected AUCs = 0.966). However, SEMO-only showed a more favorable calibration profile and lower prediction error. Although the Combined model incorporated two conventional CT-derived predictors, nodule size and spiculation, it provided limited incremental discrimination over SEMO-only. This pattern was supported by patient-level OOB aggregated analysis, in which SEMO-only and Combined retained comparably high AUC values with substantially overlapping 95% CIs: 0.905 (95% CI, 0.837–0.956) and 0.921 (95% CI, 0.859–0.966), respectively, whereas the Baseline model only reached an AUC of 0.683 (95% CI, 0.576–0.785). The stepwise decline from apparent (0.996) to optimism-corrected (0.966) and to patient-level out-of-bag AUC (0.905) is expected in a small, high-dimensional setting. We therefore regard the out-of-bag estimate as the more realistic indicator of generalizable discrimination and the optimism-corrected value as an upper bound. This residual gap reinforces that the present results are hypothesis-generating and contingent on external validation. These findings demonstrate that PBL-derived SEMO network features carry the majority of predictive information within this surgical cohort. To assess the potential clinical applicability of the SEMO-based models, we implemented an exploratory two-threshold scheme that anchored a rule-out cut-point at 98% sensitivity and a rule-in cut-point at 90% specificity. This approach identified a low-risk (0.37) subset in which nearly all malignancies were retained above the rule-out threshold, and a high-risk (0.87) subset achieving high specificity with few benign nodules misclassified, together partitioning the cohort into low, intermediate, and high-risk strata that, if externally validated, could map onto guideline-based nodule-management pathways. These findings suggest that the peripheral blood leukocyte SEMO network signature reflects malignancy-associated epigenomic reprogramming rather than nonspecific systemic inflammation, and that it may support both rule-out and rule-in decisions, potentially sparing selected patients from unnecessary invasive procedures while flagging high-risk nodules for expedited intervention. **Supplementary Table S1** summarizes the derivation population, predictors, and reported discrimination of the models most relevant to this study, together with the performance of the four comparator models when applied unchanged to our cohort.

The six retained hub–pathway features in the SEMO-only model likely orchestrate a coordinated, systemic epigenetic network pattern reflecting early-stage malignancy. The positively weighted ITCH- and PRKACA-related modules link higher malignancy risk to coordinated networks governing proteostasis, immune regulation (56), and cAMP/PKA-mediated signaling (57). In contrast, the negatively weighted AR-, LPAR2-, HDAC3-, and ITPR1-related modules under multivariable adjustment mapped to endocrine pathways (58, 59), lipid-mediated networks (60, 61), chromatin-regulation (62–64), and calcium-signaling-related cascades (65). Rather than demonstrating direct causal or protective biological mechanisms, these leukocyte-derived signatures represent a highly coordinated multi-systemic host response to early oncogenesis. By shifting from tumor DNA to circulating leukocytes, the SEMO framework effectively aggregates stochastic epigenetic cues into interpretable diagnostic pathways spanning proteostasis, motility, and immunoregulation. While these leukocyte-derived signatures naturally support biological hypothesis generation, their therapeutic utility remains strictly exploratory pending longitudinal or tissue-level validation.

The higher accuracy of the SEMO-based models over the traditional models likely arises partly because the latter were established in populations that differ fundamentally from ours. The Mayo (11), VA (12), and Brock (13) models were built on Western cohorts composed mainly of older patients and smokers with solid nodules; in the Brock development cohort, solid nodules accounted for 78.9% and non-solid/ground-glass nodules for only 15.8%. For context, other Chinese-derived cohorts also differ substantially in composition: the PKUPH (14) model was derived from larger surgically resected nodules (mean diameter 19 mm) without density stratification, and the cohort of Liang et al. (23, 24) was 45.6% solid and 34.2% part-solid, with ground-glass opacity accounting for only 20.2%. However, the epidemiological and radiological characteristics of our cohort were markedly different, being predominantly non-smoking, ground-glass–predominant (77.9%), and enriched for subcentimeter nodules in an Asian population. Consequently, the risk factors on which the traditional models rely most heavily, smoking history, spiculation, and solid density, carry little discriminative information in our setting, which plausibly explains their low AUCs and underscores the need for a model tailored to this clinically challenging subgroup, as addressed by our peripheral-blood-leukocyte DNA-methylation SEMO model. The discrimination in subcentimeter nodules may partly reflect SEMO’s network-level design, Each SEMO feature summarizes promoter methylation across an entire interacting gene set rather than a single gene, which may dampen gene-level measurement noise and constrain features to biologically coherent modules. Whether this design confers greater robustness than gene-level modeling in small, high-dimensional cohorts, however, remains to be tested directly and externally validated. The SEMO model holds promise as a peripheral blood-based adjunct for pre-operative risk-stratification tool following LDCT identification of indeterminate pulmonary nodules, complementing rather than replacing radiological assessment.

Despite these promising findings, several limitations must be acknowledged (1): the retrospective, single-center design and modest sample size necessitate prospective, large-scale multicenter validation (2); this cohort included only patients with CT-detected nodules highly suspicious for malignancy undergoing surgical resection. The malignancy prevalence of 82.6% matches the target preoperative risk re-stratification scenario but is higher than that of LDCT-screened nodule populations; thus, the model’s predictive performance cannot be extrapolated to this group (3); the lack of leukocyte sub-fraction deconvolution prevents us from isolating cell-type-specific epigenetic patterns, potentially leaving the model susceptible to residual confounding driven by systemic immune heterogeneity (66, 67); and (4) the limited number of benign cases (n = 26) relative to the number of model predictors yields a low events-per-variable ratio, which we mitigated, but cannot fully eliminate, through elastic-net penalization and the 1-SE rule (5). The reduction of the 19,832 candidate hub–pathway features to the 500 features constituting the modeling feature space was performed once across the full cohort; the internal-validation estimates consequently retain an additional optimism of undetermined magnitude that the bootstrap correction cannot recover (6). Infectious and granulomatous nodules were excluded by design, so the benign spectrum evaluated here is narrower than the spectrum met in practice. Future research will focus on validating the SEMO framework in prospective, demographically balanced multicenter cohorts and incorporating cellular deconvolution algorithms to resolve specific immune-cell-type contributions and explore whether pairing the SEMO platform with advanced radiomics or broader multi-omics architectures can further augment diagnostic performance. Nonetheless, this study highlights a crucial paradigm shift in indeterminate pulmonary nodule management, propelling clinical workflow away from passive, purely morphological observation toward biomarker-driven precision medicine.

## Data Availability

The de-identified 500 SEMO feature values and clinical–radiological variables used for model development and internal validation are provided in Supplementary Data. Further inquiries can be directed to the corresponding authors.

## Author dislaimer

The composition of the 500-gene promoter panel and the capture-probe sequences are proprietary to DeepoMe Limited.
