# Supplementary material for: Discriminating malignancy of pulmonary nodules using a peripheral blood leukocyte DNA methylation-based SEMO network model
Source: Front Oncol. 2026 Oct 28;16:1968970. doi: 10.3389/fonc.2026.1968970 (PMC13751369; doi:10.3389/fonc.2026.1968970)
Supplement: Supplementary file 1 [file DataSheet1.docx]

**Supplementary Material**

# 1 **Supplementary Tables**

**Supplementary Table S1. Prediction models for pulmonary nodule malignancy most relevant to the present study.**

| **Model (year)** | **Derivation population** | **Predictors** | **Discrimination reported in the source publication** | **AUC in the present cohort, all nodules ≤30 mm / ≤10 mm** |
| --- | --- | --- | --- | --- |
| Mayo (1997) | Single US centre; solitary indeterminate nodules 4–30 mm | Age, smoking, extrathoracic cancer >5 years earlier, nodule diameter, spiculation, upper-lobe location | AUC 0.83 | 0.695 / 0.593 |
| Veterans Affairs (2007) | US veterans with solitary pulmonary nodules | Age, pack-years of smoking, nodule diameter, years since quitting | AUC 0.79 | 0.606 / 0.469 |
| Brock / PanCan (2013) | Canadian lung-cancer screening cohort, first screening CT | Age, sex, family history of lung cancer, emphysema, nodule size, nodule type, upper-lobe location, nodule count, spiculation | AUC 0.94 (full model) | 0.748 / 0.668 |
| PKUPH (2011) | Chinese patients with surgically resected solitary pulmonary nodules, mean diameter 19 mm | Age, nodule diameter, spiculation, calcification, family history of cancer | AUC 0.888 | 0.655 / 0.458 |
| Three-gene sputum/plasma panel | Early NSCLC versus benign nodules | CDO1, SOX17, HOXA7 methylation | AUC 0.88 (sensitivity 90%, specificity 71%); 0.94 with clinical variables; performance falls below 1 cm | Not evaluated |
| Nine-locus cfDNA model | Chinese cohort | 9 cfDNA methylation loci | AUC 0.816; stage IA sensitivity 75.0% | Not evaluated |
| PulmoSeek (2021) | Chinese multicentre cohort | 100 cfDNA methylation features | Independent validation AUC 0.843; 0.878 in ground-glass nodules; in 6–10 mm nodules sensitivity 1.000 but specificity 0.250 | Not evaluated |
| PulmoSeek Plus (2023) | Chinese multicentre cohort with external validation | PulmoSeek cfDNA methylation plus 9 clinical and 58 CT features | Validation AUC ~0.91; at fixed specificity 0.50, sensitivity 0.98; sensitivity 0.99 in 5–10 mm nodules | Not evaluated |
| Integrative cfDNA + LDCT model (2024) | Two prospectively collected, retrospectively analysed multicentre cohorts | 40 cfDNA methylation markers selected from a 263-region panel, plus age and 5 CT features | External validation AUC 0.89; two-threshold strategy at 98% sensitivity and 90% specificity (cut-points 0.22 and 0.94) | Not evaluated |
| SEMO-only (present study) | 149 Chinese surgical candidates with nodules ≤30 mm; malignancy 82.6%; 77.9% ground-glass | Peripheral blood leukocyte SEMO hub–pathway network features | Internal validation only: apparent AUC 0.996, optimism-corrected 0.966, patient-level out-of-bag 0.905 | 0.966 / 0.920 (optimism-corrected) |

*Discrimination reported in the source publication refers to the derivation or principal validation cohort of that publication and is not directly comparable across rows, because the populations differ in malignancy prevalence, nodule size distribution, nodule density and smoking prevalence. AUC values in the present cohort for the Mayo, Veterans Affairs, Brock and PKUPH models were obtained by applying the original published coefficients without refitting (Supplementary Tables S9 and S10). cfDNA, cell-free DNA; LDCT, low-dose computed tomography; NSCLC, non-small cell lung cancer; PKUPH, Peking University People's Hospital; SEMO, Selective Epigenomic Module Optimization.*

**Supplementary Table S2. Bootstrap feature-selection stability and top-weighted recurrence of SEMO features in the SEMO-only model**

| **Rank** | **SEMO feature** | **Non-zero selection count, n/1000** | **Non-zero selection frequency** | **Top-weighted recurrence count, n/1000** | **Top-weighted recurrence frequency** | **Mean \|top-weight\|** | **Dominant sign** |
| --- | --- | --- | --- | --- | --- | --- | --- |
| 1 | AR__HP_ABNORMALITY_OF_LONG_BONE_MORPHOLOGY | 997 | 99.7% | 723 | 72.3% | 0.185 | − |
| 2 | ITCH__GO_UBIQUITIN_LIKE_PROTEIN_LIGASE_BINDING | 997 | 99.7% | 721 | 72.1% | 0.201 | + |
| 3 | PRKACA__GO_POSITIVE_REGULATION_OF_LOCOMOTION | 996 | 99.6% | 715 | 71.5% | 0.197 | + |
| 4 | LPAR2__GO_CELL_GROWTH | 983 | 98.3% | 598 | 59.8% | 0.180 | − |
| 5 | BIRC3__GO_POSITIVE_REGULATION_OF_TRANSFERASE_ACTIVITY | 980 | 98.0% | 455 | 45.5% | 0.171 | + |
| 6 | HDAC3__GO_CHROMATIN_BINDING | 990 | 99.0% | 447 | 44.7% | 0.167 | − |
| 7 | GATA3__GO_POSITIVE_REGULATION_OF_HEMOPOIESIS | 936 | 93.6% | 403 | 40.3% | 0.171 | − |
| 8 | HDAC3__GO_TRANSCRIPTION_COREGULATOR_BINDING | 942 | 94.2% | 378 | 37.8% | 0.177 | − |
| 9 | STAT2__GO_IMMUNE_RESPONSE_REGULATING_SIGNALING_PATHWAY | 906 | 90.6% | 327 | 32.7% | 0.175 | + |
| 10 | ITPR1__GO_CATION_TRANSMEMBRANE_TRANSPORT | 929 | 92.9% | 255 | 25.5% | 0.158 | − |

*Non-zero selection count is the number of bootstrap-derived elastic-net models in which the feature had a non-zero coefficient. Top-weighted recurrence count is the number of bootstrap sampling-detail records in which the feature appeared among recorded high-weight predictors. Mean |top-weight| was calculated only when the feature appeared in the top-weighted list. Bold type identifies one of the six predictors retained in the locked full-cohort SEMO equations.*

**Supplementary Table S3. Bootstrap feature-selection stability and top-weighted recurrence of SEMO features in the Combined model**

| **Rank** | **SEMO feature** | **Non-zero selection count, n/1000** | **Non-zero selection frequency** | **Top-weighted recurrence count, n/1000** | **Top-weighted recurrence frequency** | **Mean \|top-weight\|** | **Dominant sign** |
| --- | --- | --- | --- | --- | --- | --- | --- |
| 1 | ITCH__GO_UBIQUITIN_LIKE_PROTEIN_LIGASE_BINDING | 991 | 99.1% | 694 | 69.4% | 0.141 | + |
| 2 | PRKACA__GO_POSITIVE_REGULATION_OF_LOCOMOTION | 988 | 98.8% | 632 | 63.2% | 0.134 | + |
| 3 | AR__HP_ABNORMALITY_OF_LONG_BONE_MORPHOLOGY | 991 | 99.1% | 593 | 59.3% | 0.124 | − |
| 4 | ITPR1__GO_CATION_TRANSMEMBRANE_TRANSPORT | 966 | 96.6% | 411 | 41.1% | 0.115 | − |
| 5 | HDAC3__GO_CHROMATIN_BINDING | 976 | 97.6% | 372 | 37.2% | 0.115 | − |
| 6 | BIRC3__GO_POSITIVE_REGULATION_OF_TRANSFERASE_ACTIVITY | 945 | 94.5% | 358 | 35.8% | 0.116 | + |
| 7 | HDAC3__GO_TRANSCRIPTION_COREGULATOR_BINDING | 922 | 92.2% | 325 | 32.5% | 0.119 | − |
| 8 | LPAR2__GO_CELL_GROWTH | 884 | 88.4% | 289 | 28.9% | 0.116 | − |
| 9 | ERBB2__GO_TRANSMEMBRANE_RECEPTOR_PROTEIN_SERINE_THREONINE_KINASE_SIGNALING_PATHWAY | 843 | 84.3% | 249 | 24.9% | 0.121 | + |
| 10 | HDAC1__GO_CHROMATIN_BINDING | 933 | 93.3% | 205 | 20.5% | 0.108 | − |

*Non-zero selection count is the number of bootstrap-derived elastic-net models in which the feature had a non-zero coefficient. Top-weighted recurrence count is the number of bootstrap sampling-detail records in which the feature appeared among recorded high-weight predictors. Mean |top-weight| was calculated only when the feature appeared in the top-weighted list. Bold type identifies one of the six predictors retained in the locked full-cohort SEMO equations.*

**Supplementary Table S4. Apparent and classic bootstrap optimism-corrected performance estimates**

| **Model** | **Metric** | **Apparent estimate (95% CI)** | **Classic bootstrap optimism-corrected estimate (95% CI)** | **Estimated optimism** |
| --- | --- | --- | --- | --- |
| Baseline | AUC | 0.754 (0.655–0.844) | 0.724 (0.633–0.819) | 0.030 |
| Baseline | PR-AUC | 0.930 (0.879–0.972) | 0.920 (0.893–0.953) | 0.010 |
| Baseline | Brier score | 0.160 (0.138–0.180) | 0.160 (0.159–0.164) | +0.000 |
| SEMO-only | AUC | 0.996 (0.988–1.000) | 0.966 (0.944–0.986) | 0.030 |
| SEMO-only | PR-AUC | 0.999 (0.998–1.000) | 0.992 (0.987–0.997) | 0.007 |
| SEMO-only | Brier score | 0.036 (0.025–0.049) | 0.065 (0.051–0.080) | +0.029 |
| Combined | AUC | 0.993 (0.978–1.000) | 0.966 (0.943–0.984) | 0.027 |
| Combined | PR-AUC | 0.999 (0.995–1.000) | 0.992 (0.985–0.997) | 0.007 |
| Combined | Brier score | 0.053 (0.041–0.066) | 0.076 (0.062–0.094) | +0.023 |

*Abbreviations: AUC, area under the receiver operating characteristic curve; CI, confidence interval; PR-AUC, precision-recall area under the curve; SEMO, Selective Epigenomic Module Optimization. Apparent performance was calculated after full-cohort refitting. Classic bootstrap optimism-corrected estimates were obtained by subtracting mean optimism for AUC and PR-AUC and adding optimism for Brier score, for which lower values are better.*

**Supplementary Table S5. Composition of the exploratory risk groups and operating characteristics of the two cut-points.**

| **Characteristic** | **Low risk**  **(<0.37)** | **Intermediate risk**  **(0.37–0.87)** | **High risk**  **(≥0.87)** | **All patients** |
| --- | --- | --- | --- | --- |
| Patients, n (%) | 11 (7.4) | 52 (34.9) | 86 (57.7) | 149 (100.0) |
| Benign, n | 9 | 15 | 2 | 26 |
| Malignant, n | 2 | 37 | 84 | 123 |
| Observed malignancy rate, % (95% CI) | 18.2 (5.1–47.7) | 71.2 (57.7–81.7) | 97.7 (91.9–99.4) | 82.6 (75.7–87.8) |
| Cut-point defining the group boundary | Rule-out, 0.37 | — | Rule-in, 0.87 | — |
| Cut-point, bootstrap median (95% CI) | 0.373 (0.273–0.552) | — | 0.889 (0.723–0.963) | — |
| Sensitivity of the cut-point, % (95% CI) | 98.4 (94.3–99.6) | — | 68.3 (59.6–75.9) | — |
| Specificity of the cut-point, % (95% CI) | 34.6 (19.4–53.8) | — | 92.3 (75.9–97.9) | — |
| Positive predictive value, % (95% CI) | 87.7 (81.2–92.2) | — | 97.7 (91.9–99.4) | — |
| Negative predictive value, % (95% CI) | 81.8 (52.3–94.9) | — | 38.1 (27.1–50.4) | — |
| False negatives, n | 2 | — | 39 | — |
| False positives, n | 17 | — | 2 | — |

*Risk groups are defined on the patient-level out-of-bag predicted probabilities of the SEMO-only model. The low- and high-risk groups are bounded by the rule-out cut-point of 0.37 and the rule-in cut-point of 0.87, respectively; the operating characteristics in the lower part of the table therefore apply to those two cut-points and are entered in the corresponding columns. An em dash indicates that the quantity is not defined for that column. Applying the rule-out cut-point classifies 138 patients (92.6%) as intermediate or high risk, and applying the rule-in cut-point classifies 86 (57.7%) as high risk. Proportions are given with Wilson 95% confidence intervals. Cut-point medians and their intervals are derived from 2,000 stratified bootstrap resamples in which both cut-points were re-estimated at the same sensitivity and specificity anchors (98% and 90%). CI, confidence interval.*

**Supplementary Table S6. Operating characteristics of the SEMO-only model at alternative cut-points spanning the bootstrap 95% intervals of the rule-out and rule-in cut-points.**

| **Cut-point range** | **Cut-point** | **Below cut-point, n** | **TP** | **FN** | **TN** | **FP** | **Sensitivity, % (95% CI)** | **Specificity, % (95% CI)** | **PPV, % (95% CI)** | **NPV, % (95% CI)** |
| --- | --- | --- | --- | --- | --- | --- | --- | --- | --- | --- |
| Rule-out range | 0.27 | 4 | 123 | 0 | 4 | 22 | 100.0 (97.0–100.0) | 15.4 (6.2–33.5) | 84.8 (78.1–89.8) | 100.0 (51.0–100.0) |
| Rule-out range | 0.30 | 7 | 122 | 1 | 6 | 20 | 99.2 (95.5–99.9) | 23.1 (11.0–42.1) | 85.9 (79.2–90.7) | 85.7 (48.7–97.4) |
| Rule-out range | 0.37 (proposed) | 11 | 121 | 2 | 9 | 17 | 98.4 (94.3–99.6) | 34.6 (19.4–53.8) | 87.7 (81.2–92.2) | 81.8 (52.3–94.9) |
| Rule-out range | 0.45 | 15 | 119 | 4 | 11 | 15 | 96.7 (91.9–98.7) | 42.3 (25.5–61.1) | 88.8 (82.4–93.1) | 73.3 (48.0–89.1) |
| Rule-out range | 0.55 | 20 | 117 | 6 | 14 | 12 | 95.1 (89.8–97.7) | 53.8 (35.5–71.2) | 90.7 (84.4–94.6) | 70.0 (48.1–85.5) |
| Rule-in range | 0.72 | 33 | 109 | 14 | 19 | 7 | 88.6 (81.8–93.1) | 73.1 (53.9–86.3) | 94.0 (88.1–97.0) | 57.6 (40.8–72.8) |
| Rule-in range | 0.80 | 45 | 100 | 23 | 22 | 4 | 81.3 (73.5–87.2) | 84.6 (66.5–93.8) | 96.2 (90.5–98.5) | 48.9 (35.0–63.0) |
| Rule-in range | 0.87 (proposed) | 63 | 84 | 39 | 24 | 2 | 68.3 (59.6–75.9) | 92.3 (75.9–97.9) | 97.7 (91.9–99.4) | 38.1 (27.1–50.4) |
| Rule-in range | 0.90 | 65 | 82 | 41 | 24 | 2 | 66.7 (57.9–74.4) | 92.3 (75.9–97.9) | 97.6 (91.7–99.3) | 36.9 (26.2–49.1) |
| Rule-in range | 0.96 | 85 | 63 | 60 | 25 | 1 | 51.2 (42.5–59.9) | 96.2 (81.1–99.3) | 98.4 (91.7–99.7) | 29.4 (20.8–39.8) |

*Cut-points of 0.27–0.55 and 0.72–0.96 cover the bootstrap 95% intervals of the rule-out (0.273–0.552) and rule-in (0.723–0.963) cut-points. Positive denotes a predicted probability at or above the cut-point. 95% CIs by the Wilson method. PPV and NPV are computed at the cohort prevalence of 82.6% (123 malignant, 26 benign). TP, true positive; FN, false negative; TN, true negative; FP, false positive; PPV, positive predictive value; NPV, negative predictive value.*

**Supplementary Table S7. Operating characteristics and net benefit of the two SEMO-only cut-points at the observed and at assumed malignancy prevalences.**

| **Cut-point** | **Prevalence, %** | **Sensitivity, % (95% CI)** | **Specificity, % (95% CI)** | **PPV, % (95% CI)** | **NPV, % (95% CI)** | **Threshold probability*** | **Net benefit (95% CI)** | **Net benefit, treat all** | **Net benefit gain vs best default (95% CI)** |
| --- | --- | --- | --- | --- | --- | --- | --- | --- | --- |
| Rule-out (0.37) | 5 | 98.4 (94.3–99.6) | 34.6 (19.4–53.8) | 7.3 (5.6–9.5) | 99.8 (98.9–99.9) | 0.006 | 0.045 (0.043–0.047) | 0.044 | 0.001 (0.000–0.003) |
| Rule-out (0.37) | 10 | 98.4 (94.3–99.6) | 34.6 (19.4–53.8) | 14.3 (11.2–18.1) | 99.5 (97.8–99.9) | 0.014 | 0.090 (0.087–0.093) | 0.088 | 0.003 (−0.001 to 0.006) |
| Rule-out (0.37) | 15 | 98.4 (94.3–99.6) | 34.6 (19.4–53.8) | 21.0 (16.7–26.0) | 99.2 (96.5–99.8) | 0.021 | 0.135 (0.130–0.140) | 0.131 | 0.004 (−0.001 to 0.009) |
| Rule-out (0.37) | 20 | 98.4 (94.3–99.6) | 34.6 (19.4–53.8) | 27.3 (22.1–33.2) | 98.8 (95.1–99.7) | 0.030 | 0.181 (0.173–0.187) | 0.175 | 0.005 (−0.002 to 0.011) |
| Rule-out (0.37) | 25 | 98.4 (94.3–99.6) | 34.6 (19.4–53.8) | 33.4 (27.5–39.9) | 98.5 (93.6–99.6) | 0.040 | 0.226 (0.217–0.233) | 0.219 | 0.007 (−0.002 to 0.014) |
| Rule-out (0.37) | 30 | 98.4 (94.3–99.6) | 34.6 (19.4–53.8) | 39.2 (32.8–46.1) | 98.0 (91.9–99.5) | 0.051 | 0.271 (0.260–0.280) | 0.263 | 0.008 (−0.003 to 0.017) |
| Rule-out (0.37) | 70 | 98.4 (94.3–99.6) | 34.6 (19.4–53.8) | 77.8 (72.6–82.3) | 90.1 (67.7–97.5) | 0.225 | 0.632 (0.607–0.653) | 0.613 | 0.019 (−0.006 to 0.040) |
| Rule-out (0.37) | 82.6 (observed) | 98.4 (94.3–99.6) | 34.6 (19.4–53.8) | 87.7 (81.2–92.2) | 81.8 (52.3–94.9) | 0.370 | 0.745 (0.716–0.770) | 0.723 | 0.022 (−0.007 to 0.047) |
| Rule-out (0.37) | 90 | 98.4 (94.3–99.6) | 34.6 (19.4–53.8) | 93.1 (91.1–94.7) | 70.3 (35.2–91.2) | 0.528 | 0.812 (0.780–0.840) | 0.788 | 0.024 (−0.008 to 0.052) |
| Rule-in (0.87) | 5 | 68.3 (59.6–75.9) | 92.3 (75.9–97.9) | 31.8 (10.9–64.0) | 98.2 (97.7–98.7) | 0.069 | 0.029 (0.019–0.036) | −0.021 | 0.029 (0.019–0.036) |
| Rule-in (0.87) | 10 | 68.3 (59.6–75.9) | 92.3 (75.9–97.9) | 49.7 (20.6–79.0) | 96.3 (95.2–97.2) | 0.136 | 0.057 (0.039–0.072) | −0.041 | 0.057 (0.039–0.072) |
| Rule-in (0.87) | 15 | 68.3 (59.6–75.9) | 92.3 (75.9–97.9) | 61.0 (29.2–85.6) | 94.3 (92.6–95.6) | 0.200 | 0.086 (0.058–0.109) | −0.062 | 0.086 (0.058–0.109) |
| Rule-in (0.87) | 20 | 68.3 (59.6–75.9) | 92.3 (75.9–97.9) | 68.9 (36.8–89.4) | 92.1 (89.8–93.9) | 0.261 | 0.115 (0.077–0.145) | −0.083 | 0.115 (0.077–0.145) |
| Rule-in (0.87) | 25 | 68.3 (59.6–75.9) | 92.3 (75.9–97.9) | 74.7 (43.7–91.8) | 89.7 (86.8–92.1) | 0.320 | 0.144 (0.097–0.181) | −0.104 | 0.144 (0.097–0.181) |
| Rule-in (0.87) | 30 | 68.3 (59.6–75.9) | 92.3 (75.9–97.9) | 79.2 (50.0–93.5) | 87.2 (83.7–90.0) | 0.377 | 0.172 (0.116–0.217) | −0.124 | 0.172 (0.116–0.217) |
| Rule-in (0.87) | 70 | 68.3 (59.6–75.9) | 92.3 (75.9–97.9) | 95.4 (84.5–98.7) | 55.5 (48.5–62.3) | 0.767 | 0.402 (0.271–0.507) | −0.290 | 0.402 (0.271–0.507) |
| Rule-in (0.87) | 82.6 (observed) | 68.3 (59.6–75.9) | 92.3 (75.9–97.9) | 97.7 (91.9–99.4) | 38.1 (27.1–50.4) | 0.870 | 0.474 (0.319–0.597) | −0.342 | 0.474 (0.319–0.597) |
| Rule-in (0.87) | 90 | 68.3 (59.6–75.9) | 92.3 (75.9–97.9) | 98.8 (95.5–99.7) | 24.4 (19.6–30.0) | 0.927 | 0.517 (0.348–0.651) | −0.373 | 0.517 (0.348–0.651) |

*Sensitivity and specificity are observed values (Wilson 95% CIs) and are held constant across prevalences. PPV and NPV at assumed prevalences were computed from Bayes’ theorem, with 95% CIs by the method of Mercaldo et al.; at the observed prevalence they are the observed proportions with Wilson CIs. *Threshold probability at which the cut-point operates after the model intercept is updated by log[odds(assumed prevalence)/odds(0.826)]; the same patients fall on either side of the cut-point at every prevalence. Net benefit is evaluated at that threshold; its 95% CI and that of the gain over the better of treat-all and treat-none come from 2,000 stratified bootstrap resamples (seed 42). Rule-out: probability <0.37 classified as low risk; rule-in: probability ≥0.87 classified as high risk. n = 149 (123 malignant, 26 benign). PPV, positive predictive value; NPV, negative predictive value.*

**Supplementary Table S8. Expected clinical impact of the two cut-points per 1,000 patients at the observed and at assumed malignancy prevalences.**

| **Cut-point** | **Prevalence, %** | **Classified by the cut-point, per 1,000 (95% CI)** | **Correctly classified, per 1,000 (95% CI)†** | **Misclassified, per 1,000 (95% CI)‡** |
| --- | --- | --- | --- | --- |
| Rule-out (<0.37) | 5 | 329.7 (182.7–512.4) | 328.8 (182.7–511.5) | 0.8 (0.0–2.0) |
| Rule-out (<0.37) | 10 | 313.2 (173.1–486.2) | 311.5 (173.1–484.6) | 1.6 (0.0–4.1) |
| Rule-out (<0.37) | 15 | 296.7 (163.5–460.1) | 294.2 (163.5–457.7) | 2.4 (0.0–6.1) |
| Rule-out (<0.37) | 20 | 280.2 (153.8–434.0) | 276.9 (153.8–430.8) | 3.3 (0.0–8.1) |
| Rule-out (<0.37) | 25 | 263.7 (144.2–407.9) | 259.6 (144.2–403.8) | 4.1 (0.0–10.2) |
| Rule-out (<0.37) | 30 | 247.2 (134.6–381.8) | 242.3 (134.6–376.9) | 4.9 (0.0–12.2) |
| Rule-out (<0.37) | 70 | 115.2 (63.2–173.1) | 103.8 (57.7–161.5) | 11.4 (0.0–28.5) |
| Rule-out (<0.37) | 82.6 (observed) | 73.8 (40.3–114.1) | 60.4 (33.6–94.0) | 13.4 (0.0–33.6) |
| Rule-out (<0.37) | 90 | 49.2 (23.1–78.9) | 34.6 (19.2–53.8) | 14.6 (0.0–36.6) |
| Rule-in (≥0.87) | 5 | 107.2 (32.5–216.8) | 34.1 (30.1–38.2) | 73.1 (0.0–182.7) |
| Rule-in (≥0.87) | 10 | 137.5 (65.0–241.4) | 68.3 (60.1–76.4) | 69.2 (0.0–173.1) |
| Rule-in (≥0.87) | 15 | 167.8 (97.6–265.9) | 102.4 (90.2–114.6) | 65.4 (0.0–163.5) |
| Rule-in (≥0.87) | 20 | 198.1 (130.1–290.4) | 136.6 (120.3–152.8) | 61.5 (0.0–153.8) |
| Rule-in (≥0.87) | 25 | 228.4 (162.6–315.0) | 170.7 (150.4–191.1) | 57.7 (0.0–144.2) |
| Rule-in (≥0.87) | 30 | 258.7 (195.1–339.5) | 204.9 (180.4–229.3) | 53.8 (0.0–134.6) |
| Rule-in (≥0.87) | 70 | 501.1 (432.5–563.7) | 478.0 (421.0–535.0) | 23.1 (0.0–57.7) |
| Rule-in (≥0.87) | 82.6 (observed) | 577.2 (503.4–644.3) | 563.8 (496.5–630.9) | 13.4 (0.0–33.6) |
| Rule-in (≥0.87) | 90 | 622.3 (545.2–692.0) | 614.6 (541.3–687.8) | 7.7 (0.0–19.2) |

*†Rule-out: benign nodules classified as low risk, i.e. resections that could be avoided. Rule-in: malignant nodules classified as high risk. ‡Rule-out: malignant nodules classified as low risk (missed cancers). Rule-in: benign nodules classified as high risk (benign resections). Counts are the product of prevalence and the observed sensitivity or specificity; 95% CIs are percentiles of 2,000 stratified bootstrap resamples (seed 42). The lower bound of 0.0 for misclassified counts reflects resamples with no misclassification in the 26 benign or 2 low-risk malignant patients and indicates the imprecision of these estimates.*

**Supplementary Table S9. Contextual same-cohort diagnostic performance: nodule diameter ≤30 mm**

| **Model** | **AUC (95% CI)** | **Sensitivity (95% CI)** | **Specificity (95% CI)** | **Youden index (95% CI)** | **PPV (95% CI)** | **NPV (95% CI)** | **Accuracy (95% CI)** |
| --- | --- | --- | --- | --- | --- | --- | --- |
| Mayo | 0.695 (0.594–0.796) | 56.4% (47.3–64.6) | 84.6% (66.5–93.8) | 0.410 (0.214–0.540) | 95.2% (86.7–97.8) | 26.5% (20.0–40.0) | 60.8% (53.1–68.5) |
| VA | 0.606 (0.493–0.719) | 48.6% (40.1–57.5) | 76.9% (57.9–89.0) | 0.255 (0.054–0.414) | 91.9% (81.6–95.8) | 21.7% (16.2–34.3) | 53.0% (45.7–61.5) |
| Brock | 0.748 (0.657–0.839) | 47.1% (38.6–55.9) | 92.3% (75.9–97.9) | 0.395 (0.225–0.503) | 97.1% (88.6–99.1) | 24.5% (18.8–37.0) | 54.3% (47.0–62.8) |
| PKUPH | 0.655 (0.548–0.762) | 48.6% (40.1–57.5) | 84.6% (66.5–93.8) | 0.332 (0.143–0.468) | 94.4% (85.0–97.5) | 23.4% (17.8–36.1) | 54.2% (47.0–62.8) |
| SEMO-only | 0.966 (0.944–0.986) | 87.8% (80.9–92.5) | 80.8% (62.1–91.5) | 0.686 (0.495–0.811) | 95.6% (90.1–98.1) | 58.3% (42.2–72.9) | 86.6% (80.2–91.1) |
| Combined | 0.966 (0.943–0.984) | 87.8% (80.9–92.5) | 88.5% (71.0–96.0) | 0.763 (0.588–0.860) | 97.3% (92.4–99.1) | 60.5% (44.7–74.4) | 87.9% (81.7–92.2) |

*AUC, area under the receiver operating characteristic curve; PPV, positive predictive value; NPV, negative predictive value. SEMO-only and Combined AUC confidence intervals were taken from bootstrap optimism correction. For contextual classical-model comparisons, AUC 95% CIs were estimated using the Hanley-McNeil approximation. Confidence intervals for classification measures were estimated from observed case counts using Wilson/binomial-based intervals. Classical models were not locally recalibrated.*

**Supplementary Table S10. Contextual same-cohort diagnostic performance: nodule diameter ≤10 mm**

| **Model** | **AUC (95% CI)** | **Sensitivity (95% CI)** | **Specificity (95% CI)** | **Youden index (95% CI)** | **PPV (95% CI)** | **NPV (95% CI)** | **Accuracy (95% CI)** |
| --- | --- | --- | --- | --- | --- | --- | --- |
| Mayo | 0.593 (0.466–0.720) | 32.9% (23.6–43.9) | 91.3% (73.2–97.6) | 0.242 (0.054–0.371) | 92.9% (77.4–98.0) | 28.4% (19.4–39.5) | 46.1% (36.7–55.7) |
| VA | 0.469 (0.333–0.605) | 53.2% (42.3–63.8) | 56.6% (36.8–74.4) | 0.097 (-0.132–0.312) | 80.8% (68.1–89.2) | 26.0% (15.9–39.6) | 53.9% (44.3–63.3) |
| Brock | 0.668 (0.551–0.785) | 58.2% (47.2–68.5) | 69.6% (49.1–84.4) | 0.278 (0.047–0.467) | 86.8% (75.2–93.5) | 32.7% (21.2–46.6) | 60.8% (51.1–69.7) |
| PKUPH | 0.458 (0.322–0.594) | 15.2% (8.9–24.7) | 100.0% (85.7–100.0) | 0.152 (0.033–0.231) | 100.0% (75.8–100.0) | 25.6% (17.7–35.4) | 34.3% (25.8–43.9) |
| SEMO-only | 0.920 (0.855–0.970) | 84.8% (75.3–91.1) | 87.0% (67.9–95.5) | 0.718 (0.517–0.833) | 95.7% (88.1–98.5) | 62.5% (45.3–77.1) | 85.3% (77.1–90.9) |
| Combined | 0.931 (0.870–0.977) | 87.3% (78.2–93.0) | 91.3% (73.2–97.6) | 0.786 (0.602–0.881) | 97.2% (90.3–99.2) | 67.7% (50.1–81.4) | 88.2% (80.6–93.1) |

*AUC, area under the receiver operating characteristic curve; PPV, positive predictive value; NPV, negative predictive value. SEMO-only and Combined AUC confidence intervals were taken from bootstrap optimism correction. For contextual classical-model comparisons, AUC 95% CIs were estimated using the Hanley-McNeil approximation. Confidence intervals for classification measures were estimated from observed case counts using Wilson/binomial-based intervals. Classical models were not locally recalibrated.*

**Supplementary Figures**

**Supplementary Figure S1. Prevalence dependence of the predictive values and of net benefit.**

*(A) Negative predictive value and (B) positive predictive value of the SEMO-only model at the rule-out cut-point of 0.37 (sensitivity 98.4%, specificity 34.6%) and the rule-in cut-point of 0.87 (sensitivity 68.3%, specificity 92.3%), plotted against the malignancy prevalence of the target population with sensitivity and specificity held at their observed values. The dashed line marks the 82.6% prevalence of the present surgical cohort and the shaded band the 5–30% range plausible for LDCT-detected indeterminate nodules. As prevalence falls the rule-out cut-point becomes more informative and the rule-in cut-point substantially less so. (C) Decision curve for the SEMO-only model after updating the intercept to an assumed prevalence of 20%, shown against the treat-all and treat-none references; net benefit remains above both across the threshold range. Numerical values are given in Supplementary Table S7.*

**Supplementary Figure S2. Decision curves at assumed and observed malignancy prevalences.**

*Net benefit of the Baseline, SEMO-only and Combined models at assumed prevalences of 5–30% (A–F), representing lower-prevalence CT-detected populations, at 70% and 90% (G, I), the range reported for resected nodules, and at the observed prevalence of 82.6% (H). At assumed prevalences each model intercept was updated by log[odds(assumed prevalence)/odds(0.826)], which shifts predicted probabilities without changing discrimination; panel H uses the out-of-bag predicted probabilities unchanged. Circles and triangles mark the net benefit of the SEMO-only model at the rule-out (0.37) and rule-in (0.87) cut-points, plotted at the threshold probability at which each cut-point operates after recalibration (Supplementary Table S7). Dashed line, treat all; dotted line, treat none. The y-axis scale differs between panels. n = 149 (123 malignant, 26 benign).*
